# A New Strategy for Silver Deposition on Au Nanoparticles with the Use of Peroxidase-Mimicking DNAzyme Monitored via a Localized Surface Plasmon Resonance Technique

**DOI:** 10.3390/s17040849

**Published:** 2017-04-13

**Authors:** Joanna Kosman, Jacqueline Jatschka, Andrea Csaki, Wolfgang Fritzsche, Bernard Juskowiak, Ondrej Stranik

**Affiliations:** 1 Laboratory of Bioanalytical Chemistry, Faculty of Chemistry, Adam Mickiewicz University, Umultowska 89b, 61-614 Poznan, Poland; juskowia@amu.edu.pl; 2 Department of Nano Biophotonics, Leibniz Institute of Photonic Technology (IPHT), 07745 Jena, Germany; jjatschka88@gmail.com (J.J.); andrea.csaki@leibniz-ipht.de (A.C.); wolfgang.fritzsche@leibniz-ipht.de (W.F.); ondrej.stranik@leibniz-ipht.de (O.S.)

**Keywords:** DNAzyme, silver deposition, LSPR, localized surface plasmon resonance, Au nanoparticles, AuNPs

## Abstract

Peroxidase-mimicking DNAzyme was applied as a catalyst of silver deposition on gold nanoparticles. This DNAzyme is formed when hemin binds to the G-quadruplex-forming DNA sequence. Such a system is able to catalyze a redox reaction with a one- or two-electron transfer. The process of silver deposition was monitored via a localized surface plasmon resonance technique (LSPR), which allows one to record scattering spectrum of a single nanoparticle. Our study showed that DNAzyme is able to catalyze silver deposition. The AFM experiments proved that DNAzyme induced the deposition of silver shells of approximately 20 nm thickness on Au nanoparticles (AuNPs). Such an effect is not observed when hemin is absent in the system. However, we noticed non-specific binding of hemin to the capture oligonucleotides on a gold NP probe that also induced some silver deposition, even though the capture probe was unable to form G-quadruplex. Analysis of SEM images indicated that the surface morphology of the silver layer deposited by DNAzyme is different from that obtained for hemin alone. The proposed strategy of silver layer synthesis on gold nanoparticles catalyzed by DNAzyme is an innovative approach and can be applied in bioanalysis (LSPR, electrochemistry) as well as in material sciences.

## 1. Introduction

DNAzymes are specific DNA oligonucleotides, which possess catalytic activity [1]. The search for novel catalytic systems different from protein enzymes was dictated by drawbacks of popular enzymes. DNAzymes in comparison with proteins are more thermally stable, simpler in synthesis and purification, and can hybridize with complementary strands. One of the most attracting systems nowadays is a DNAzyme with peroxidase activity, which can catalyze the reaction between H_2_O_2_ and suitable substrate [2]. To form DNAzyme, DNA oligonucleotide must first adopt G-quadruplex structure and create complex with hemin. Most of the bioassays based on this system use two kinds of substrates: luminol (chemiluminescence readout) or ABTS (colorimetric signal) [3]. The progress in DNAzyme-related studies is advancing in two directions. The first approach involves the modification of DNAzyme in order to enhance peroxidase activity. Researchers developed probes with covalently attached hemin as well as probes with possessing terminal adenines or d (CCC) flanks that gave promising results [4,5,6]. The second trend in this field is focused on the development of new methods or analytical platforms, as well as more efficient substrates for peroxidase reaction [7,8,9,10].

The catalytic reaction of silver deposition is well known for systems involving horseradish peroxidase (HRP) [11,12]. So far, no attempts for transferring this reaction for DNAzyme system have been undertaken. Potentially, if successful, this reaction can be used for the development of new bioassays based on such detection techniques as surface plasmon resonance and electrochemistry combined with microscopy. A non-contact sensitive detection of silver deposition can be also afforded by the localized surface plasmon resonance because the Ag shell around AuNP changes dramatically light scattering spectrum of the NP [13,14]. Therefore, even small peroxidase activity of the DNAzyme can be detected via localized plasmon resonance scattering (LSPR). To transfer the reaction of silver deposition to the DNAzyme system, fundamental studies should be carried out including characterization and optimization of the reaction system as well as its implementation to the LSPR format. In this communication, we show that DNAzyme based on the PS2.M sequence can be used for silver deposition on gold nanoparticles. The LSPR technique was successfully exploited to demonstrate the DNAzyme activity and performance of the developed system.

## 2. Materials and Methods

### 2.1. Materials

All DNA oligonucleotides (HPLC purity) were purchased from Generi Biotech (Hradec Kralove, Czech Republic) and used without further purification. The sequences of oligonucleotides used in this study are gathered in Table 1.

Gold nanoparticles (80 nm or 100 nm diameters) and Silver Enhancing Kit for Optical and Electron Microscopy were purchased from BBI Solutions (Cardiff, UK). All other chemicals were purchased from Sigma-Aldrich (St. Louis, MO, USA) and used without further purification. Hemin stock solution was prepared in DMSO and stored in dark at −30 °C no longer than 1 month.

### 2.2. Instruments

All LSPR measurements were recorded on AxioImager Z1m optical microscope (Carl Zeiss, Jena, Germany) in dark field geometry (Objective 100×, NA = 0.75). The light source was a tungsten halogen lamp with a continuous spectrum at 3200 K. The use of dark field configuration allowed for the collection of only scattered light, which passed through a pinhole (150 µm diameter) to the Acton Research SpectraPro 2300i microspectrometer (Princeton Instruments, Trenton, NJ, USA) with a grating with 150 lines per mm and a Peltier-cooled CCD camera.

### 2.3. Sensor Preparation Procedure

LSPR measurements were performed on borosilicate glass plates with chrome pattern microstructure. Chips before usage were cleaned successively with organic solutions (acetone, rotisol, and ethanol) and water for 10 min each in ultrasonic bath. Next, slides were activated by oxygen plasma etching (2 × 6 min, 50 W, 5 Pa), silanized with APES [14] for 10 min, and then washed with water in an ultrasonic bath for 5 min. Gold nanoparticles (80 nm or 100 nm diameter) were immobilized on a glass slide by incubation with a solution of 1.2 × 10^8^ NPs/mL for 1 h. Thiol-modified oligonucleotides were immobilized on active AuNP surfaces by incubation overnight (16 h) in a Tris–HCl buffer (DNA at concentration of 1 µM). In the next step, chips were subjected to 6-mercaptohexanol blocking (1 mM MCH in water) for 1 h. The MCH interacted with unbound active sites on the nanoparticle surface. This treatment prevented the non-specific binding of other molecules to the nanoparticle surface [13].

### 2.4. LSPR Measurement Procedure

The spectra of selected nanoparticles (usually 10 NPs) were recorded after each step of procedure. Besides NP spectra (*I_NP_*), also background (*I_BG_*), light source (*I_LS_*), and dark current (*I_NL_*) signals were recorded. The spectra were normalized and corrected for the background, dark current, and light source using Equation (1).

$$I_{nor=\;\frac{I_{cor}}{\max\left(I_{cor}\right)}}$$

where

(1) $$I_{cor}=\;\frac{I_{NP}-I_{BG}}{I_{LS}-I_{NL}}.$$

In the first stage, the scattering spectra of analyzed NPs were recorded before DNA oligonucleotide immobilization. The effect of oligonucleotide immobilization (16 h, 1 µM DNA oligonucleotide) was also followed by scattering signal measurement. Next, the chip was transferred back to a small Petri dish and blocked with MCH (30 min incubation) to eliminate free binding sites. The chip, after washing, was transferred to the microscope to record subsequent scattering spectra. Depending on the system design, the next step involved hybridization with G-quadruplex oligonucleotide or creation of G-quadruplex-hemin complex on the initially immobilized oligonucleotide. Hybridization with G4 oligonucleotide (1 µM) was conducted for 2 h in a Tris–HCl buffer. DNAzyme was generated by the incubation of G-quadruplex on a chip with 2 µM hemin for 30 min. The silver deposition reaction was performed for 20 s using a silver enhancing kit. The reaction was stopped by washing the chip with water; after that, scattering spectra of NPs were recorded.

### 2.5. Scanning Electron Microscopy (SEM) Measurement

The immobilized nanoparticles were characterized after the different steps of experiment with a scanning electron microscope JEOL 6700F (Joel, Japan).

### 2.6. Atomic Force Microscopy (AFM) Measurement

The morphology of the analyzed nanoparticle was measured by an atomic force microscope Dimension TM 3100 (Digital Instruments, Veeco, NY, USA). The further analysis of obtained images was realized with Gwyddion 2.31 software.

## 3. Results

**Scheme 1:**
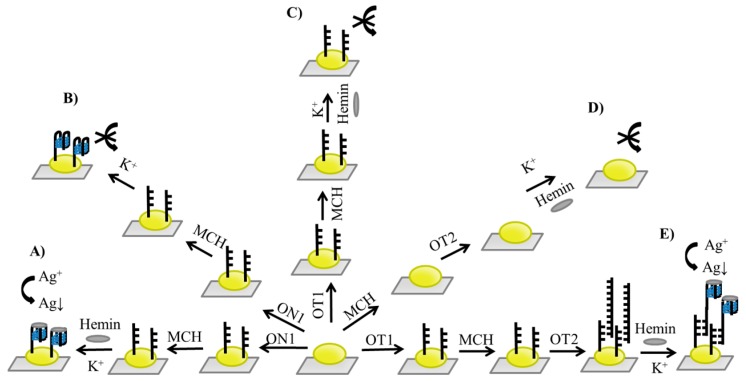
Scheme of experimental setup showing Ag deposition experiments (Routes A and E) and control experiments (Routes B, C, and D): (**A**) Detection system with DNAzyme-based ON1 probe immobilized on AuNP. (**B**) Control experiment for Route A (no hemin). (**C**) Negative control (for Route E) with OT1 capture probe but without the OT2 DNAzyme probe addition. (**D**) Second negative control (for Route E) in the absence of OT1 capture probe but with OT2 G-quadruplex oligonucleotide. (**E**) Ag deposition system based on the OT1/OT2 hybridization approach.

The design of experimental setup is depicted on Scheme 1. Two Ag deposition protocols (A and E) as well as three reference experiments (B, C, and D) were carried out. The first system involved immobilization of ON1 oligonucleotide bearing the PS2.M sequence, which, after forming G-quadruplex in the presence of potassium ion, can bind hemin and catalyze the deposition of silver on AuNPs (Route A). The second Ag deposition system involved hybridization of the Au immobilized probe (OT1) with OT2 oligonucleotide containing a domain complementary to OT1 and G-quadruplex domain with a telomeric sequence that can generate DNAzyme with hemin (Route E). Three reference experiments were designed to clarify whether silver deposition can be observed in the presence of PS2.M probe: without hemin (Route B), in the presence of hemin and an immobilized OT1 probe that was unable to form G-quadruplex (Route C), and with the addition of DNAzyme-forming probe OT2 to AuNPs that were not modified by immobilization of oligonucleotide (Route D).

**Figure 1:**
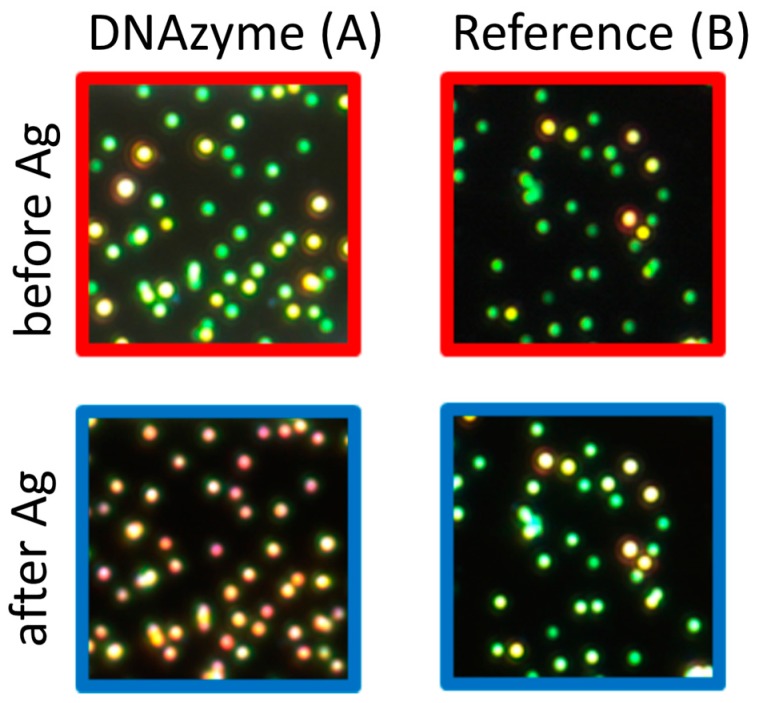
Left panels—Dark field images of NPs with attached DNAzyme (Scheme 1, Route **A**) before (**top**) and after (**bottom**) silver enhancement experiment. Right panels—Dark field images of NPs without DNAzyme presence (Scheme 1, Route **B**) before (**top**) and after (**bottom**) silver enhancement experiment.

**Figure 2:**
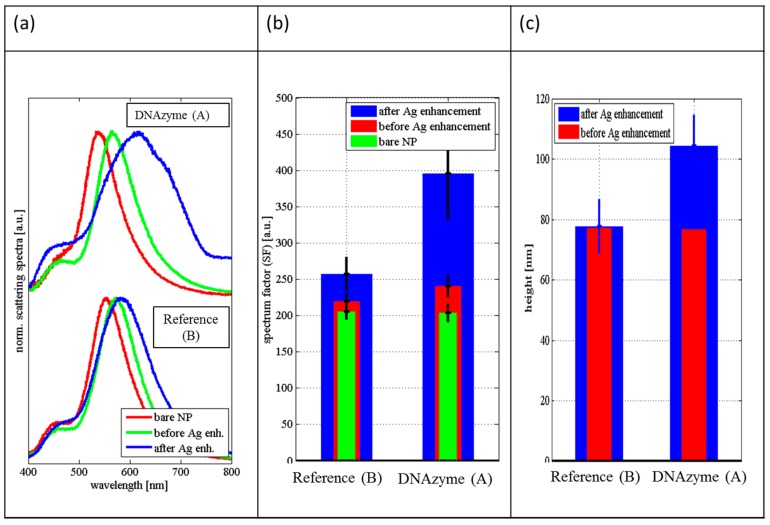
Spectral and microscopic characterization of the Ag deposition on AuNPs for experiments A and B (Scheme 1). Panel (**a**) shows normalized scattering spectra of two representative NPs during the consecutive steps of silver enhancement procedure for NP with attached DNAzyme (top) and for NP without DNAzyme (bottom). Bare NP (red line), NP just before Ag enhancement reaction (green line), after Ag enhancement (blue line). Scattering spectra for other NPs are shown in Figures S1 and S2 in Supplementary Materials. Panel (**b**) presents values of the spectrum factor (SF) for the spectra of the NPs with and without DNAzyme during the process of the silver deposition. Panel (**c**) shows values of the average height of NPs before Ag enhancement reaction and after Ag deposition for NPs with DNAzyme and without DNAzyme (reference probe). The height was measured by atomic force microscopy. The AFM images are shown in Figures S4 and S5 in Supplementary Materials.

We were able to follow single NPs exhibiting LSPR activity by applying the dark field illumination microscopy with color camera (Figure 1A). Obtained results showed that silver deposition on the NPs catalyzed by DNAzyme, changed the color of the NPs as expected for such a process [15]. In the control experiment (NPs without DNAzyme), the color change is not observed (Figure 1B). The fully spectrally resolved scattering spectra of the single NPs at all stages of the Ag enhancement experiments were recorded, and representative examples are shown in Figure 2 (spectra for miscellaneous particles are presented in Figures S1–S3 in Supplementary Materials). During the initial steps, in which DNA/MCH were attached to the NP, the LSPR peak position was shifted. Mercaptohexanol was used to saturate active sites on gold surfaces, and its length was similar to the C6 mercapto-linker that was used for DNA immobilization on NPs [16]. The shift in peak position indicates successful attachment of the DNA/MCH (Figure 2a). When Ag^+^ is reduced in the presence of DNAzyme, the LSPR peak of the single nanoparticle becomes very broad (Figure 2a). Such an effect is not observed for NPs without DNAzyme (Route B). This broadening can be quantified by the calculation of the “spectrum factor” (SF). The SF equals the surface below the normalized scattering spectrum. The low value of SF corresponds to a sharp peak in spectrum, while the high value of SF relates to the broad peak. As presented in Figure 2b, the SF values increase significantly after a silver enhancement reaction in the presence of DNAzyme attached to the NPs. To get more information on the morphology of the silver shells deposited on the AuNPs, we conducted AFM measurements for both systems (Figure 2c, Figures S4 and S5 in Supplementary Materials). The results showed that the thickness of the deposited Ag on the NP with immobilized DNAzyme was around 20 nm. As expected, there was no change in the height (size) after Ag enhancement reaction for NP with DNA but in the absence of hemin molecule (Route B).

**Figure 3:**
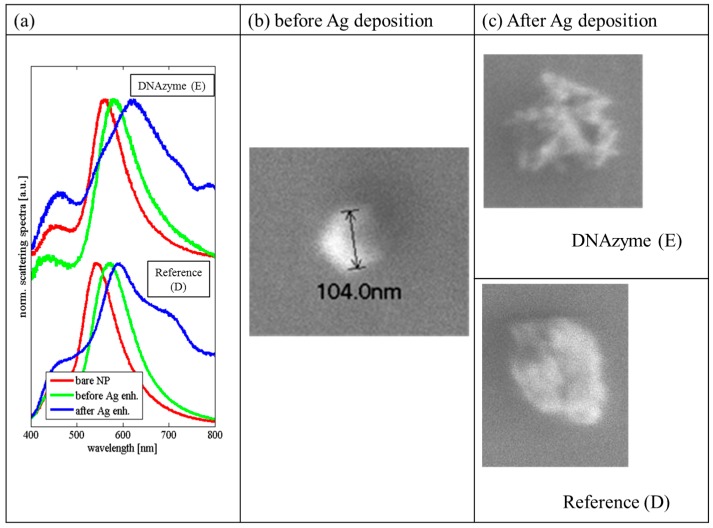
Characterization of the Ag deposition on AuNPs for Routes D and E (Scheme 1). Panel (**a**) shows normalized scattering spectra of two representative NPs during the consecutive steps of silver enhancement procedure for NP with immobilized DNAzyme (top) and for NP without DNAzyme (bottom). Bare NP (red line), NP just before Ag enhancement reaction (green line), NP after Ag enhancement (blue line). Scattering spectra for other NPs are presented in Figures S2 and S3 in Supplementary Materials). Panel (**b**) shows representative SEM image of bare NP. Panel (**c**) shows representative SEM images of NPs after silver enhancement reaction for immobilized DNAzyme (top) and for NP without DNAzyme (bottom) (more SEM images in Figure S7). In both cases, Ag is deposited on the NPs. Silver forms a star-like shell on NPs with DNAzyme, but a different, rather unstructured shell is formed on NPs without DNAzyme (incubated with hemin alone).

The performance of Ag deposition catalyzed by DNAzyme was further examined on the system based on hybridization of capture (OT1) and sensing (OT2) probes (Route E in Scheme 1). Such a setup is more compatible with future sensing applications of the system due to design flexibility (e.g., for sandwich type sensors [3]). This setup allowed us to design two other reference experiments (Routes C and D in Scheme 1) to further assess selectivity of the Ag deposition process. Route C was designed to test if hemin can bind to a single stranded OT1 oligonucleotide, and Route D was expected to show whether OT2/hemin complex could adsorb non-specifically onto the NP surface. As shown in Figure 3, some silver deposition occurred in both DNAzyme catalyzed and reference experiments. The Ag deposition was manifested by a strong broadening of LSPR peak (Figure 3a, Figures S2 and S3 in Supplementary Materials). The increase of the SF for reference experiments with hemin (Routes C and D) was similar to that observed for the DNAzyme catalyzed Ag deposition (Route E) (Figure S6 in Supplementary Materials). It should be noted that Routes C and D yielded similar Ag deposition efficiencies although in the case of Route C the PS2.M oligonucleotide responsible for DNAzyme formation was absent (only OT1 capturing single stranded oligonucleotide was adsorbed on NPs). Therefore, one can conclude that the nonspecific binding of hemin on AuNPs is responsible for catalyzing Ag deposition. It is well known that hemin possesses weak catalytic activity [2,3,17]. Concluding, the monitoring of DNAzyme activity through Ag deposition on the nanoparticle appeared to be hampered by high blank value resulted from catalytic activity of hemin nonspecifically adsorbed on AuNPs. To differentiate the silver deposition morphology, we acquired SEM images of the silver-modified AuNPs with and without DNAzyme (Routes D and E in Scheme 1) Representative examples are shown in Figure 3c and Figure S7 (Supplementary Materials). The NP with Ag deposited by DNAzyme possesses a star-like shape. On the contrary, silver on the NPs without DNAzyme (adsorbed hemin) is deposited rather uniformly, yielding an intact spherical shell. This proves that the mechanism of silver deposition should be different in these two cases. Our study on the mechanism of the silver deposition reaction catalyzed by DNAzyme was hampered by an unknown composition of the Enhancement Kit purchased from BBI Solutions. This kit probably contains hydroquinone and may include hydrogen peroxide. To prove that DNAzyme indeed catalyzes the reaction of silver deposition, we performed experiments in a bulk solution by monitoring the reaction progress using hydroquinone/benzoquinone absorption bands in the UV spectral range. Obtained results confirmed that DNAzyme was able to catalyze the reaction between silver cations and reducing agent (hydroquinone), both in the absence and presence of H_2_O_2_ (Figure S8). The reaction probably includes electron transfer from Hq to hemin Fe(III) and then the reduction of silver. A chain reaction of Ag^+^ reduction cannot be excluded, where initially deposited silver by DNAzyme catalyzes more deposition and the silver grains grow in size. As mentioned already, the most plausible reason for the high values of the negative control experiments, Routes C and D, is non-specific adsorption of hemin on the gold NPs and/or binding with single-stranded DNA oligonucleotide immobilized on NPs. The evidence of hemin binding on gold surface can be found in the literature [18]. It was also proven that hemin alone possesses redox activity [17], so it can catalyze silver deposition upon the adsorption on gold surface. Moreover, it was shown that AuNPs themselves can catalyze silver deposition [19]. Such a reaction, however, is very slow and inefficient. On the other hand, non-specific binding of OT2/hemin complex (DNAzyme) on AuNPs can be also considered as alternative explanation. Nevertheless, DNAzyme non-specifically adsorbed on NPs should produce Ag shell with morphology similar to that observed for immobilized DNAzyme. Because this is not the case (star-like versus spherical shape), adsorption of OT2/hemin complex on AuNPs should be excluded. The different morphology of silver shells obtained using DNAzymes and non-specifically bound hemin molecule has a potential for the application in material sciences.

## 4. Conclusions

We successfully conducted silver deposition on gold nanoparticles catalyzed by peroxidase-mimicking DNAzyme. For the monitoring of silver deposition process, we applied a localized surface plasmon resonance technique as well as additional AFM and SEM microscope imaging. We showed that Ag deposition on gold nanoparticles was initiated by the redox activity of DNAzyme attached to the AuNPs. The deposition of Ag resulted in the formation of an approximately 20 nm thick shell. The shell growth on NPs with specifically attached DNAzyme was highly anisotropic (star-like shape). However, non-specific adsorption of hemin on AuNPs also initiates Ag deposition. This phenomenon can be applied in material sciences for the production of silver shells with different morphologies. However, for further biosensing application of the developed DNAzyme-based Ag deposition system requires the suppression of this non-specific effect. We are currently designing new DNAzyme probes with covalently attached hemin, which should result in the exclusion of free hemin binding artifact on AuNPs. Silver deposition catalyzed by such covalent DNAzyme can be applied in sensor technology, especially for detection platforms based on SPR and electrochemical or electrical techniques. This system can also be applied in materials science for manufacturing nanoparticles covered with silver shells.

## Figures and Tables

**Table 1:** Sequences of DNA oligonucleotides used in this study (underlined DNAzyme domain).

Name	DNA Sequence
ON1	HS-(CH_2_)_6–5_’-TTTT GGGTAGGGCGGGTTGGG3’
OT1	HS-(CH_2_)_6–3_’-TTTT TTA GGC AGC TCG TCT CAA-5’
OT2	5’-AAT CCG TCG AGC AGA GTT TTA GGG TTA GGG TTA GGG TTA GGG TTA GGG TTA GGG TTA GGG TTA GGG-3’

## Supplementary Materials

The following are available online at http://www.mdpi.com/1424-8220/17/4/849/s1, Figure S1: Scattering spectra of NPs at the different stages of the Ag deposition procedure (experiments A and B in Scheme 1), Figure S2: Scattering spectra of NPs at the different stages of the Ag deposition procedure (experiments E and D in Scheme 1), Figure S3: Scattering spectra of NPs at the different stages of the detection schema C in Scheme 1, Figure S4: AFM images of nanoparticles with attached PS2.M-DNAzyme (route A in Scheme 1) before (a) and after (b) silver deposition reaction. Table summarizes heights of the nanoparticles shown in panel (a) and (b), Figure S5: AFM images of nanoparticles with attached PS2.M sequence but without hemin (negative control - route B in Scheme 1) before (a) and after (b) silver deposition reaction. Table summarizes heights of the nanoparticles before (panel (a)) and after (panel (b)) silver enhancement reaction, Figure S6: Average values of spectrum factor (SF) of spectra recorded for experiments with hybridiztion probes OT1 and OT2 (routes E and reference C, D in Scheme 1), Figure S7: SEM images of silver enhanced Au nanoparticles in case of immobilized DNAzyme and in the case of nanoparticles without the DNAzyme, Figure S8: Progress in reaction of hydroquinone oxidation to quinone in silver reduction reaction monitored at 249 nm.
