# Supplementary material for: A New Strategy for Silver Deposition on Au Nanoparticles with the Use of Peroxidase-Mimicking DNAzyme Monitored via a Localized Surface Plasmon Resonance Technique
Source: Sensors (Basel). 2017 Apr 13;17(4):849. doi: 10.3390/s17040849 (PMC5424726; doi:10.3390/s17040849)
Supplement: Supplementary file 1 [file sensors-17-00849-s001.pdf]

Supplementary Materials

New strategy for silver deposition on Au nanoparticles with the use of peroxidase-mimicking DNAzyme monitored by Localized Surface Plasmon Resonance technique

J. Kosman[1,+;§], J. Jatschka[2,+], A. Csaki[2], W. Fritzsche[2], B. Juskowiak[1] and O. Stranik[2]

Supplementary Figures

(a) – positive control	(b) – negative control
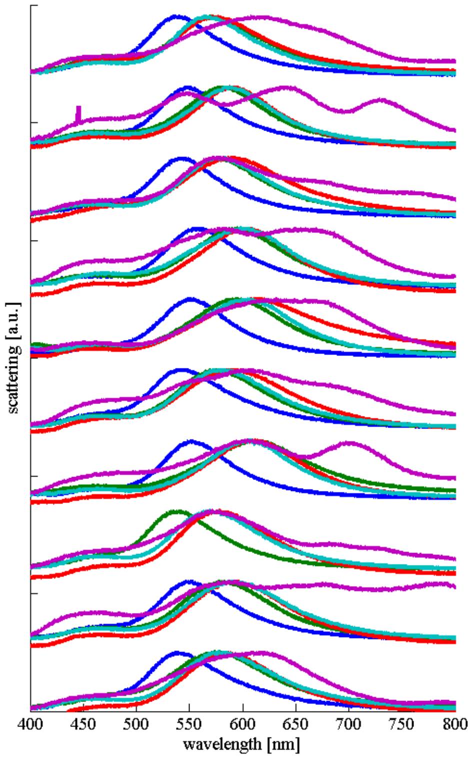	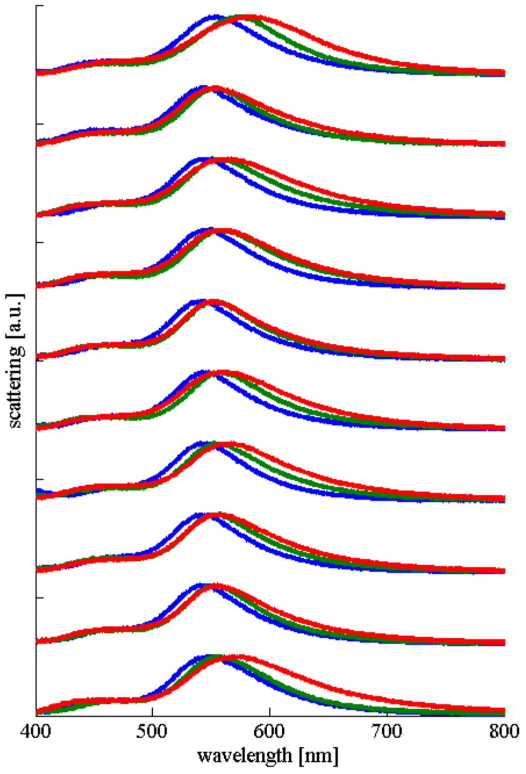

Figure S1. Scattering spectra of NPs at the different stages of the Ag deposition procedure (experiments A and B in Scheme 1). Each set of spectra represents one NP.
Panel (a) experiment A – positive control: bare NP (blue), after adsorption of ON1 DNA (green), after adsorption of MCH (red), after formation of DNAzyme -addition of K+ and hemin solution (turquoise) , after silver enhancement reaction (violet).
Panel (b)–experiment B - negative control: bare NP (blue), after adsorption of MCH (green), after silver enhancement reaction (red).

(a) – positive control	(b) – negative control

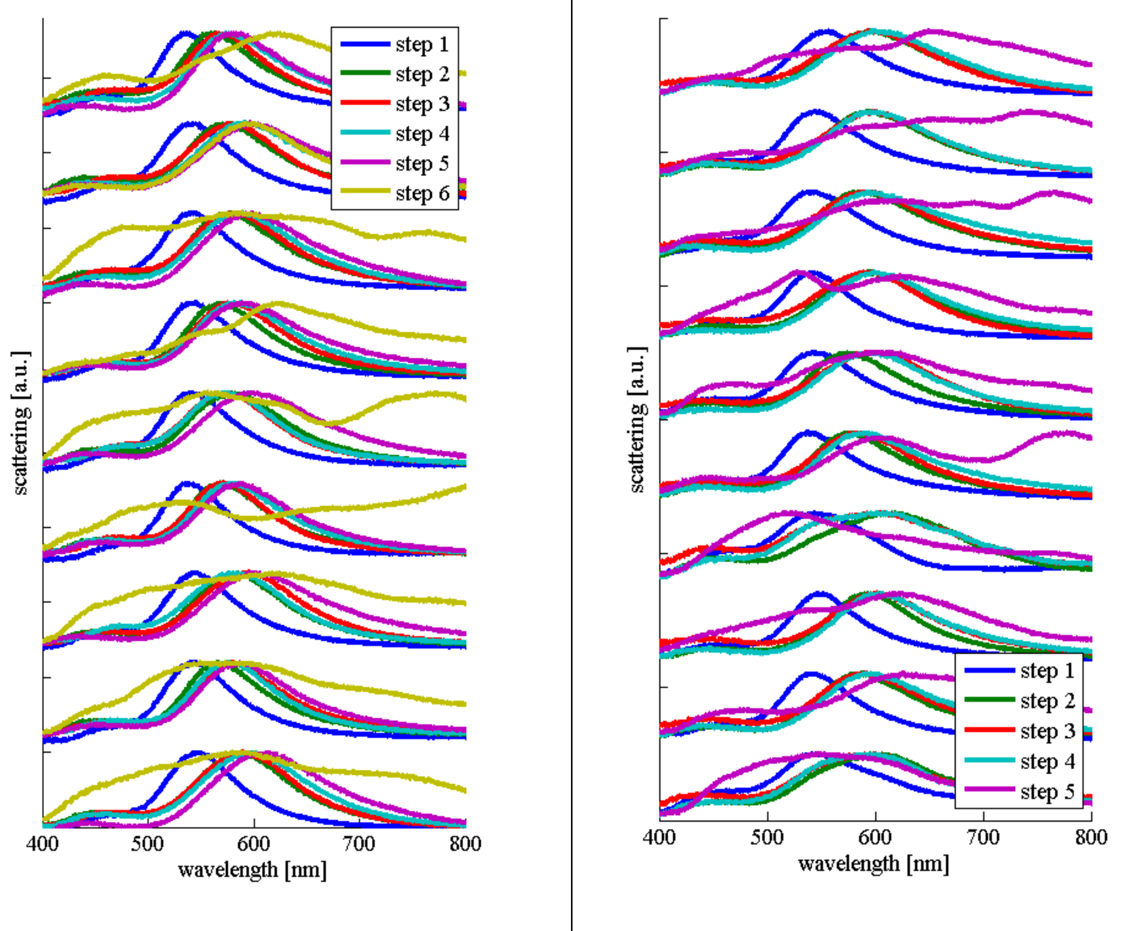

Figure S2. Scattering spectra of NPs at the different stages of the Ag deposition procedure (experiments E and D in Scheme 1). Each set of spectra represents one NP.

Panel (a) experiment E – positive control: bare NP (blue), after adsorption of OT1 DNA (green), after adsorption of MCH (red), after OT2 DNA hybridization (turquoise), after formation of DNAzyme - addition of K+ and hemin (violet), after silver enhancement reaction (yellow).

Panel (b)–experiment D - negative control: bare NP (blue), after adsorption of MCH (green), after incubation with OT2 probe (red), after addition of K+ and hemin (turquoise), after silver enhancement reaction (violet).

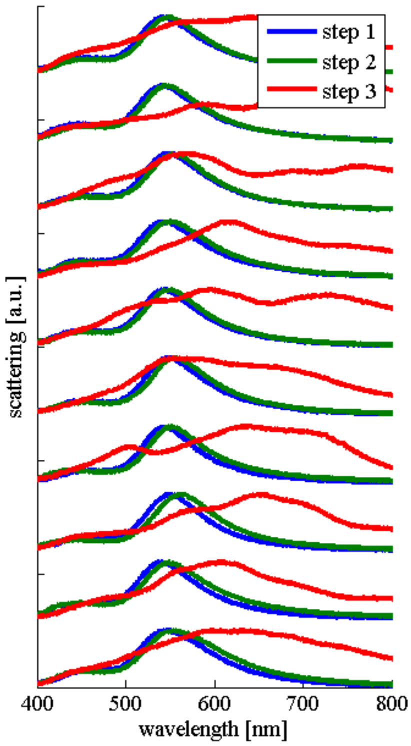

Figure S3. Scattering spectra of NPs at the different stages of the detection schema C in Scheme 1. Experiment C - negative control: bare NP (blue), after adsorption of MCH/OT1 (green), after silver enhancement reaction (red).

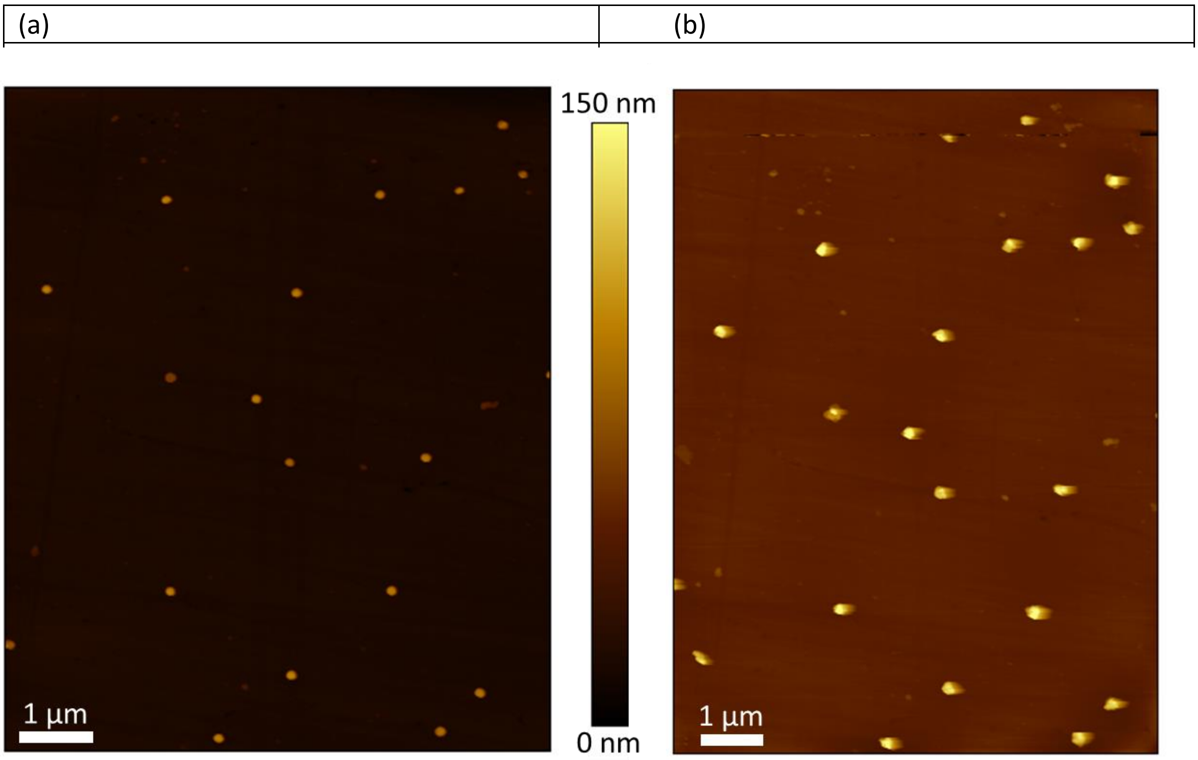

Nanoparticle	Before Ag deposition (panel (a))	After Ag deposition (pael (b))	Nanoparticle	Before Ag deposition (panel (a))	After Ag deposion (panel (b))
**NP1**	**82.3nm**	**96.4nm**	**NP8**	**85.5nm**	**100.2nm**
**NP2**	**81.1nm**	**102.5nm**	**NP9**	**75.2nm**	**112.5nm**
**NP3**	**82.2nm**	**112.1nm**	**NP10**	**81.3nm**	**115.2nm**
**NP4**	**67.7nm**	**88.5nm**	**NP11**	**81.4nm**	**114.4nm**
**NP5**	**76.4nm**	**110.8nm**	**NP12**	**75.7nm**	**85.9nm**
**NP6**	**75nm**		**NP13**	**74.5nm**	**96.8nm**
**NP7**	**82.5nm**	**116.1nm**			

Figure S4. AFM images of nanoparticles with attached PS2.M-DNAzyme (route A in Scheme 1) before (a) and after (b) silver deposition reaction. Table summarizes heights of the nanoparticles shown in panel (a) and (b).

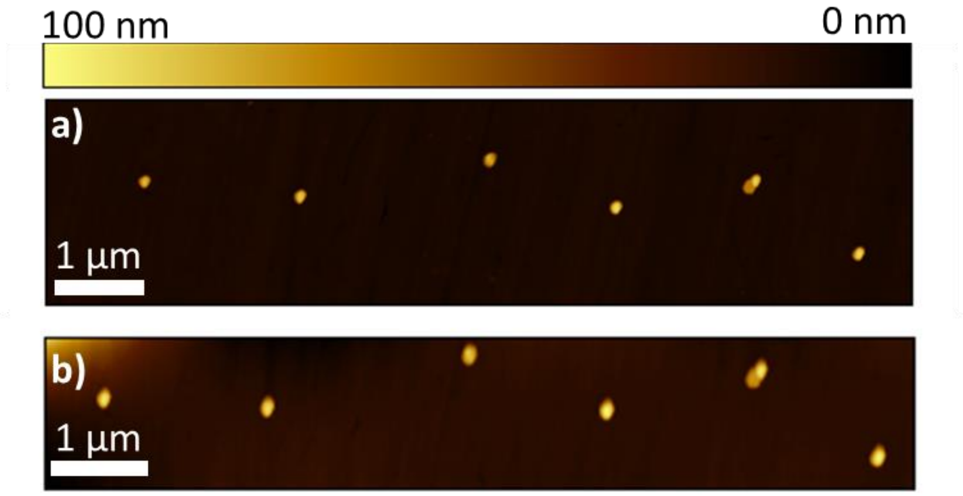

Nanoparticle	Before Ag deposition (panel (a))	After Ag deposition (panel (b))	Nanoparticle	Before Ag deposition (panel (a))	After Ag deposition (panel (b))
NP1	85.9nm	80.1nm	NP6	68.8nm	76.1nm
NP2	80nm	79.6nm	NP7	72.6nm	77.8nm
NP3	86.3nm	78.2nm	NP8	57.8nm	70.6nm
NP4	82.3nm	83nm	NP9	79.2nm	73.1nm
NP5	87nm	755nm	NP10	71.5nm	82.5nm

Figure S5.: AFM images of nanoparticles with attached PS2.M sequence but without hemin (negative control - route B in Scheme 1) before (a) and after (b) silver deposition reaction. Table summarizes heights of the nanoparticles before (panel (a)) and after (panel (b)) silver enhancement reaction.

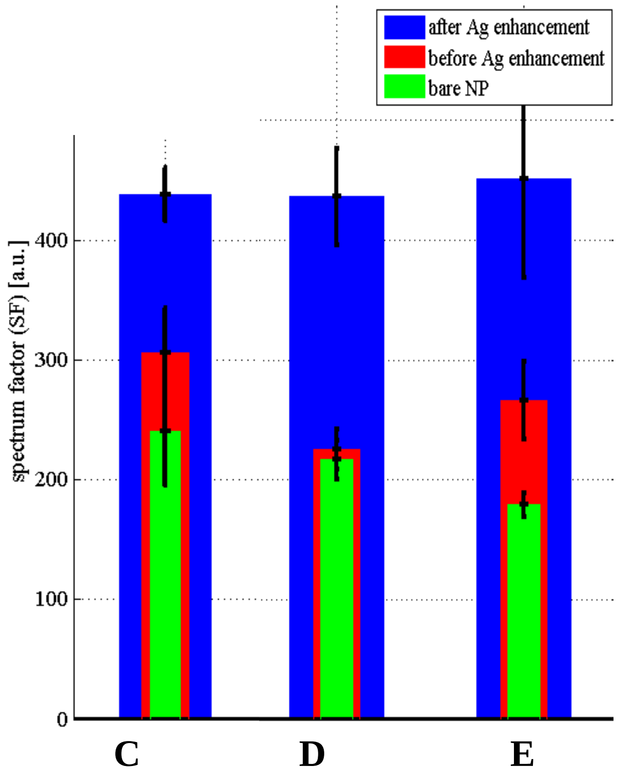

Figure S6. Average values of spectrum factor (SF) of spectra recorded for experiments with hybridiztion probes OT1 and OT2 (routes E and reference C, D in Scheme 1). Values of SF are show for the NPs without DNAzyme (reference C and D) and with DNAzyme (positive control). The SF equals the surface below the normalized spectrum. The low value of SF corresponds to sharper peak in the spectrum.

Silver depostion by DNAzyme immobilized on AuNP	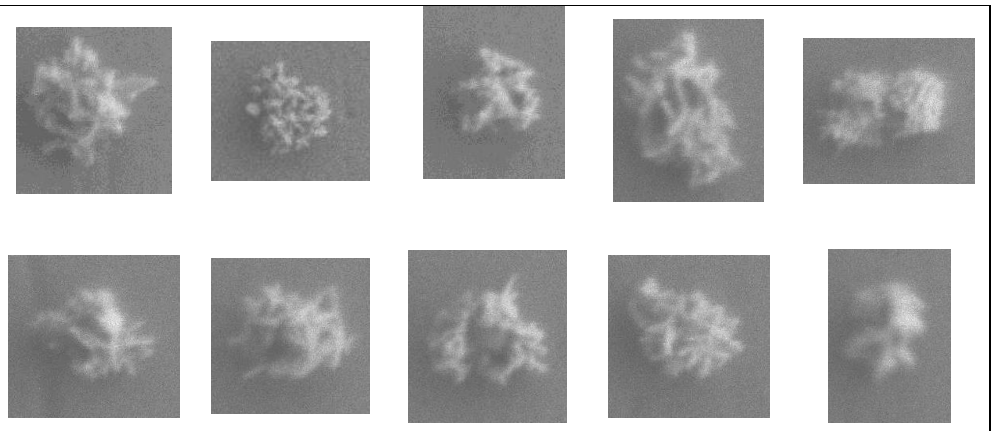
Silver deposition on blind probes	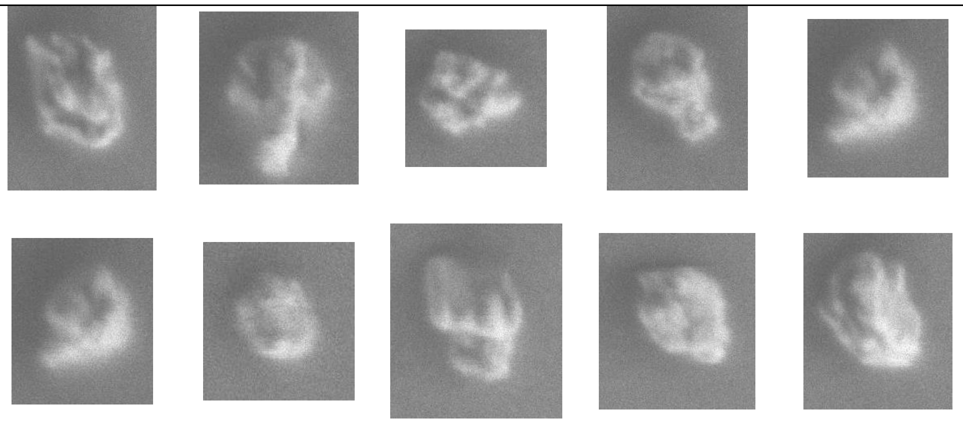

Figure S7. SEM images of silver enhanced Au nanoparticles in case of immobilized DNAzyme and in the case of nanoparticles without the DNAzyme. The Ag shell grown on NP with DNAzyme exhibits strong anisotropic star-like growth. The Ag shell grown on NP without DNAzyme does not have such a strong anisotropy.

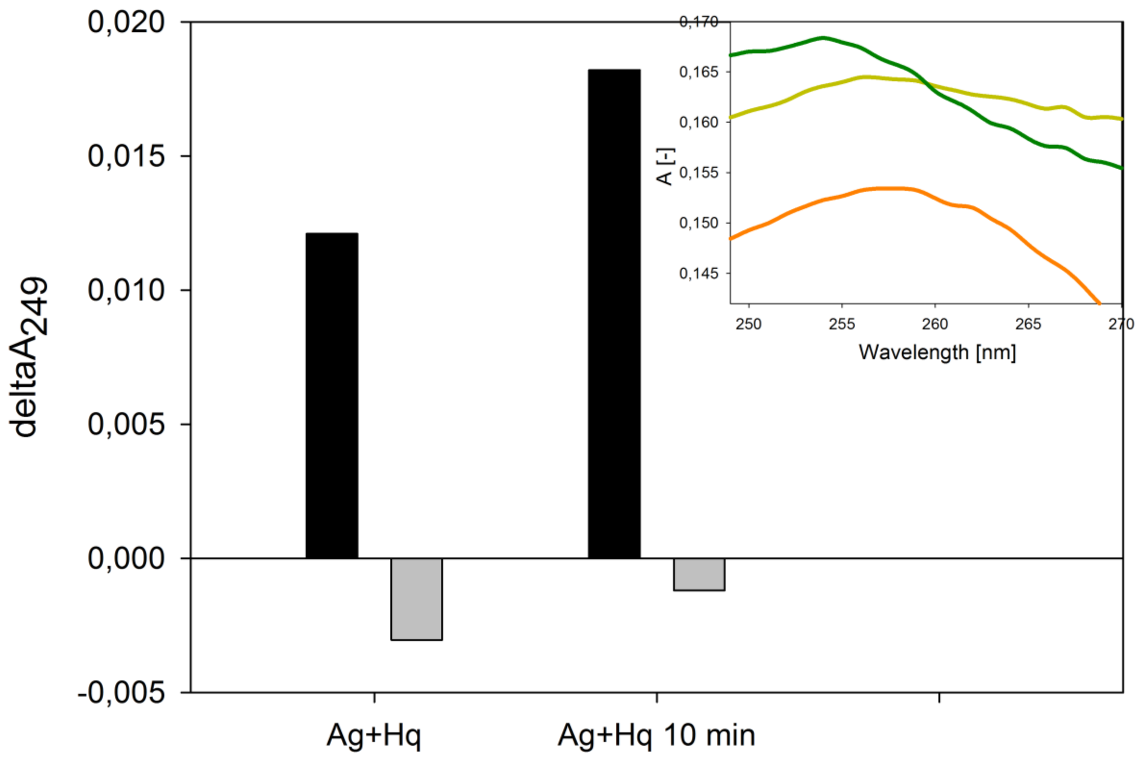

Figure S8.Progress in reaction of hydroquinone oxidation to quinone in silver reduction reaction monitored at 249 nm (black bare– DNAzyme, grey bare–reference probe without hemin). The bars corresponds to the differences between absorbance at 250 nm. The absorbance of the solution containing DNAzyme ant silver is assumed as 0. Insert: UV-Vis spectra of DNAzyme system before hydroquinone addition (orange line), 1 minute after Hq addition,(grey) and after 10 minutes (black). Conditions: 10 mM Tris-Ac, 100 mM KAc, 1 µM PS2.M, 1 µM hemin, 6 µM Hq, 6 µM $AgNO_3$.
